# Sequel and therapeutic modalities of leptospirosis associated severe pulmonary haemorrhagic syndrome (SPHS); a Sri Lankan experience

**DOI:** 10.1186/s12879-019-4094-0

**Published:** 2019-05-22

**Authors:** Nalaka Herath, Wimalasiri Uluwattage, Theshanthi Weliwitiya, Lilani Karunanayake, Sarath Lekamwasam, Neelakanthi Ratnatunga, Panduka Karunanayake, Sugeesha Wickramasinghe, Sanjitha Patabendi, Suranjith Senaviratne, Suneth Agampodi

**Affiliations:** 1 Teaching Hospital, Karapitiya, Galle, Sri Lanka; 2 Department of Bacteriology, Medical Research Institute, Colombo, Sri Lanka; 3 Department of Medicine, Faculty of Medicine, University of Ruhuna, Galle, Sri Lanka; 4 Department of Pathology, Faculty of Medicine, University of Peradeniya, Peradeniya, Sri Lanka; 5 Department of Medicine, Faculty of Medicine, University of Colombo, Colombo, Sri Lanka; 6 University College, London, UK; 7 Department of Community Medicine, Faculty of Medicine and Allied Sciences, Rajarata University of Sri Lanka, Saliyapura, Sri Lanka

**Keywords:** Leptospirosis, Severe pulmonary hemorrhagic syndrome, Sri Lanka, Therapeutic plasma exchange, Galle

## Abstract

**Background:**

The emergence of leptospirosis-associated severe pulmonary hemorrhagic syndrome (SPHS) with high case fatality has been reported from many countries. Understanding of clinical disease and sequel of SPHS needs larger studies with adequate numbers. The purpose of this study was to describe the characteristics and sequel by different therapeutic approaches for SPHS in Leptospirosis in Sri Lanka.

**Methods:**

This study was conducted at Teaching Hospital-Karapitiya (THK), Galle, Sri Lanka from June 2015 to December 2017. THK is the main tertiary care center for the Southern Province. All confirmed-cases of leptospirosis who presented during this period and were admitted to five medical units of THK were included in this study. SPHS was defined as a patient presenting; haemoptysis, arterial hypoxemia (Acute Lung Injury Score < 2.5), haemoglobin drop (10% from the previous value), or diffused alveolar shadows in the chest radiograph, without alternative explanation other than leptospirosis.

**Results:**

Of the 128 MAT confirmed cases of leptospirosis, 111 (86.7%) had acute kidney injury (AKI) whilst SPHS was seen in 80 (62.5%). Patients typically developed SPHS within the first week of illness, mostly on days 4 and 5. The case fatality rate of this study sample was 28.1% (*n* = 36), while for patients with SPHS, it was 41.5%. Most of the deaths (*n* = 19) were within the first 3 days of admission (on the same day 8, and within next 48 h 11). Among SPHS patients, 59 received therapeutic plasma exchange (TPE). The survival rate was higher (*n* = 35, 74.5%) when the TPE was performed within the first 48 h of detecting SPHS compared to patients in whom the procedure was done after 48 h (*n* = 5, 54.5%). Of the 19 leptosprosis patients with SPHS who did not receive TPE, 17 died (89.5%). However, the group of patients who received TPE was primarily the patients survived beyond day 3.

**Conclusions:**

We observed that during the study period, SPHS was common and the mortality rate was higher in the study area. The treatment modalities tested need further evaluation and confirmation.

## Background

Leptospirosis is a zoonosis caused by multiple serovars of the spirochaete *Leptospira interrogans*. It is possibly the most prevalent zoonosis in the world [1]. The estimated annual leptospirosis case load exceeds one million with more than 50,000 deaths [1]. Whilst the majority of these cases are mild or subclinical, life threatening severe leptospirosis is common in many tropical countries and the case fatality rate is typically higher than a disease like dengue. The reported organ involvement and severe complications in leptospirosis varies widely [2] and are most likely due to differences in sampling. The severe complications are often attributed to the inherent pathogenicity of the different strains or host immuno-pathological responses, whilst the exact pathophysiology of some complications are not fully ascertained as yet [3].

The emergence of leptospirosis-associated severe pulmonary hemorrhagic syndrome (SPHS) with high case fatality has been reported from many countries including Salvador, Brazil, India and some parts of South America. A case fatality rate of 74% has been reported among SPHS patients despite aggressive supportive care [3–5].

Pulmonary involvement in leptospirosis varies from subtle clinical manifestations to severe pulmonary hemorrhage and acute respiratory distress syndrome [6]. It is generally believed that neither thrombocytopenia nor a decrease in clotting factors alone is sufficient to account for the observed SPHS [7, 8]. Immunoglobulin deposition in the alveolar septum, alveolar spaces and type I and II pneumocytes, leading to pneumocyte necrosis have been observed in SPHS [9–11]. Although, the exact pathophysiological precesses are still unclear, dysregulated immune-related processes are the most plausible cause for SPHS in leptospirosis.

The standard treatment of leptospirosis includes antibiotics and supportive care. Supportive care often involves intensive care with inotropes, respiratory support and renal replacement therapy (RRT). Glucocorticoids (GC) are a commonly used adjuvant therapy for severe leptospirosis with or without lung involvement. Although it is logical to use high dose GCs to restore the dysregulated host immune response, studies have shown an increased risk of nosocomial infections associated with its use [12]. Therapeutic plasma exchange (TPE) is another adjuvant therapy which can suppress the host immune responses rapidly with a good safety profile. It also provides the missing enzymes and clotting factors since it uses fresh frozen plasma as replacement fluid. There are few studies and case reports on the successful use of TPE in SPHS [13–16].

Sri Lanka had seen an emergence of leptospirosis since 2008 [17] and has had sustained outbreak [18] with an estimated 10,423 hospitalization and 730 deaths annually [19]. During the recent years, SPHS due to leptospirosis associated with high case fatality has been reported from the southern part of Sri Lanka [20, 21]. The first well documented series of pulmonary involvement in leptospirosis was reported from Ragama in 1977 in seven cases [22]. During the 2008 Leptospirosis outbreak in Peradeniya, 60 of the 132 severe leptospirosis cases had pulmonary involvement with 30 deaths [23]. More recently, 12 of 919 suspected leptospirosis patients were reported as having lung involvement [24]. Except for the autopsy study and the 1977 case series, the focus of the previous studies were not on pulmonary involvement. The purpose of this study was to describe the characteristics and factors associated with SPHS in Leptospirosis. Furthermore, we studied the therapeutic and adverse effects of the commonly used adjuvant therapies for SPHS.

## Methods

### Study setting

This study was conducted at Teaching Hospital-Karapitiya (THK), Galle, Sri Lanka from June 2015 to December 2017. THK is the main tertiary care center for the Southern Province. The Southern province is heavily burdened with leptospirosis and previous reports show a high proportion of febrile cases to be due to leptospirosis [25] and high fatality rates to be associated with SPHS [20]. Due to the heavy burden of severe leptospirosis observed in THK, therapeutic plasma exchange (TPE) and intravenous immunoglobulin (IVIG) were offered to patients with SPHS as an alternative therapy. The decision to offer these therapies were made by the treating physician on an individual basis due to the lack of high level evidence for implementing this as part of a guideline.

All confirmed-cases of leptospirosis who presented during this period and were admitted to five medical units were included in this study. The respective medical teams headed by specialist physicians managed the patients independently. Microscopic Agglutination Test (MAT) was used to confirm the clinically suspected leptospirosis cases. MAT was performed at the Medical Research Institute, Colombo using 14 pathogenic *Leptospira* serovars (Table 1) and *L. biflexa patoc-1*. MAT positivity was defined as a titer of 320 or more, seroconversion or a four-fold rise in paired samples. The case confirmation was done as requested by the treating physicians.

**Table 1:** Serovars used in the MAT panel

	Species	Serovar	Strain	Serogroup
1.	*Leptospira interrogans*	Australis	Ballico	Australis
2.	*Leptospira interrogans*	Bankinang	Bankinang	Autamnalis
3.	*Leptospira interrogans*	Bataviae	Swart	Bataviae
4.	*Leptospira interrogans*	Bakeri	LT79	Tarassovi
5.	*Leptospira interrogans*	Rathnapura	Wumalasena	Grippotyphosa
6.	*Leptospira interrogans*	Hardjo	Hardjoprajitno	Sejroe
7.	*Leptospira interrogans*	Icterohaemorrhagiae	RGA	icterohaemorrhagiae
8.	*Leptospira interrogans*	Pyrogenes	Salinem	Pyrogenes
9.	*Leptospira interrogans*	Pomona	Pomona	Pomona
10.	*Leptospira interrogans*	Hebdomadis	Hebdomadis	Hebdomadis
11.	*Leptospira interrogans*	Cynopteri	3522C	Cynopteri
12	*Leptospira interrogans*	Canicola	Hond Uterecht IV	Canicola
13	*Leptospira interrogans*	Poi	Poi	Javanica
14	*Leptospira interrogans*	Sarmin	Sarmin	Sarmin
15	*Leptospira biflexa*	Patoc	Patoc 1	Semaranga

Clinical data were recorded by a trained research assistant using an interviewer administered questionnaire. Investigators visited patients regularly and recorded the clinical features. In addition, a data extraction sheet was used to collect investigation findings and management details from patient records.

### Leptospirosis-associated severe pulmonary haemorrhagic syndrome

Leptospirosis associated SPHS was defined as a patient with serologically confirmed leptospirosis presenting with one or more of the following; haemoptysis, arterial hypoxemia (Acute Lung Injury Score < 2.5), haemoglobin drop (10% from the previous value), or diffused alveolar shadows in the chest radiograph, without alternative explanation other than leptospirosis. Acute kidney injury (AKI) was defined as suggested in the KDIGO guidelines [26]. Myocarditis was diagnosed based on findings on ECG and echocardiography.

### Therapeutic plasma exchange

Patient’s height, weight and haemotocrit had entered to machine and the machine automatically calculates the patient’s blood volume and plasma volume. One plasma volume had been exchanged with Fresh Frozen Plasma (FFP). Percentage of replacement was kept around 90–95% as most of these patients had acute kidney injury with low urine output. Mean number of cycles were three and done on three consecutive days.

### Ethical considerations

Informed written consent was taken from all study participants. Ethical approval for the study was obtained from Ethical Review Committee, Faculty of Medicine and University of Ruhuna.

## Results

The study included 128 MAT-confirmed leptospirosis patients. Mean age of the patients was 46.2(SD 14.5). 107(83.6%) were males. The highest number of patients were from Elpitiya Medical Officer of Health (MOH) area (*n* = 22) followed by the Baddegama (*n* = 9) and Poddala (*n* = 8) regions of the Galle district, Sri Lanka. Mean duration of fever at presentation was 5.1(2.3) and the duration of hospital stay varied from 1 to 28 days with a median of 7 (IQR 4–10).

Out of 128 patients studied, 116 (90.6%) patients had at least one of the four main complications (Table 2). A majority (*n* = 111, 86.7) had acute kidney injury (AKI) whilst SPHS was seen in 80 patients (62.5%). The case fatality rate of this study sample was 28.1% with 36 deaths. The death rate was significantly higher among patients with complications compared to those without complications (Table 3). Patients with cardiac involvement showed the highest fatality rate (52% deaths). However, AKI was the main progonostic marker, as no patients died when other complications were present without AKI.

**Table 2:** Complications associated with leptospirosis

Complication	N	%
AKI	111	86.7
SPHS	80	62.5
Liver failure	43	33.6
Myocarditis	25	19.5

*AKI* acute kidney injury, *SPHS* leptospirosis associated severe pulmonary haemorrhage syndrome

**Table 3:** Mortality / survival associated with leptospirosis complications

Parameter		Died		Survived		*P* value^*^
		N	%	N	%	
SPHS	Positive	33	41.2	47	58.8	0.0001
	Negative	4	8.3	44	91.7	
AKI	Positive	36	32.4	75	67.6	0.0031
	Negative	0	0.0	17	100.0	
Liver failure	Positive	20	46.5	23	53.5	0.0034
	Negative	17	20.0	68	80.0	
Myocarditis	Positive	13	52.0	12	48.0	0.0069
	Negative	24	23.3	79	76.7	

^*^Chi-square test or Fisher’s exact test

*AKI* acute kidney injury, *SPHS* leptospirosis associated severe pulmonary haemorrhage syndrome

### Severe pulmonary Heamorrhagic Syndrom

**Fig. 1:**
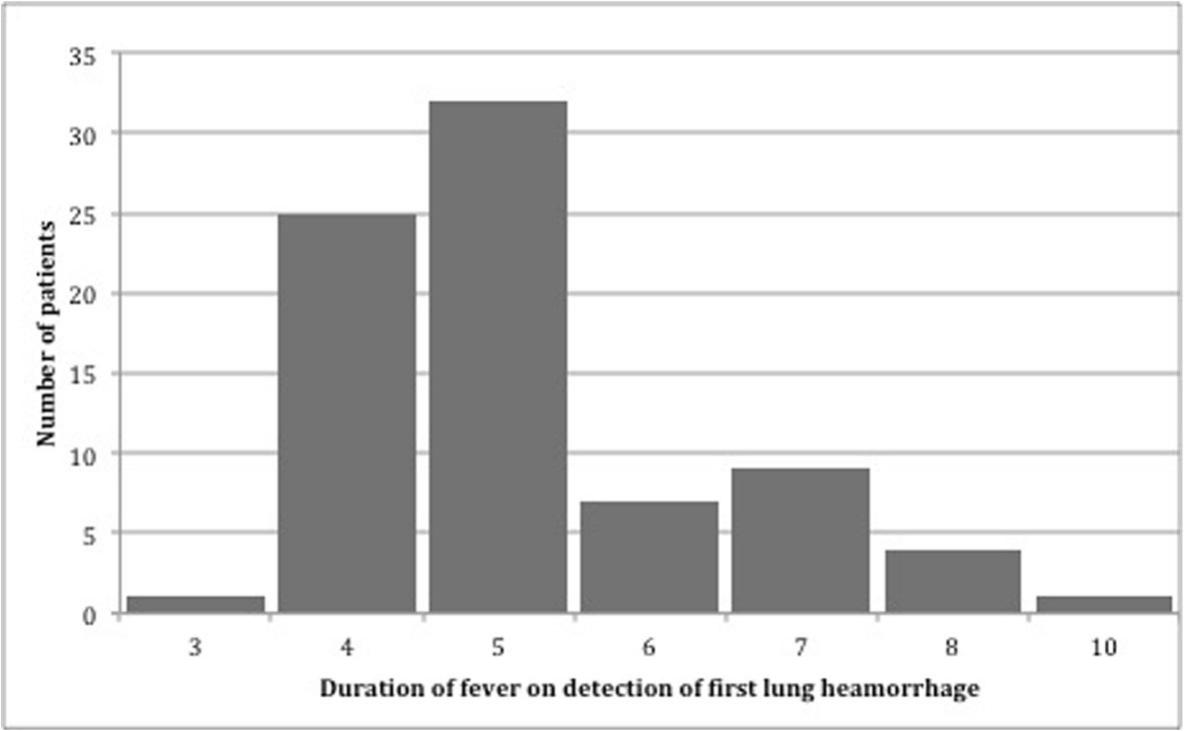
Distribution of detection of first lung haemorrhage by day of illness

Of the 80 patients who developed SPHS, complete clinical details were available from 78; 68(85%) were males, mean age: 45.6 (SD-13.2) years. Patients typically developed SPHS within the first week of illness, mostly on days 4 and 5 (Fig. 1).

All patients had progressive shortness of breath (SOB) with some patients complaining of chest pain. Cough and haemoptysis was less common and reported by 18 (23.1%) patients. On examination, most common signs were tachypnea (n 69, 88.5%), tachycardia (n 69, 88.5%), hypotension (n 61, 78.2%), and bilateral crepitations (n 73, 93.6%). Re-appearance of hypotension, tachypnoea after initial resuscitation was likelier secondary to SPHS. Milder form of lung hemorrhages would have gone unnoticed as they were asymptomatic. But in this study sample, all had one or more of following; haemoptysis, arterial hypoxemia (acute lung injury score < 2.5), haemoglobin drop (10% from the previous value), diffused alveolar shadows in the chest radiograph.

Blood biochemistry of all these patients were assessed during their hospital stay. Since the date of admission and the date of haemorrhage was variable, we observed the values on admission values, on the day prior to and after the haemorrhage. Median total white cell counts of the SPHS patients were markedly raised whilst the platelet counts were low. As expected, serum creatinine values were high in the majority [median - 261 (IQR 146–411)]. Among the patients in whom the blood gas analysis was done on day one (*n* = 44), 23 had metabolic acidosis with low PCO_2_ and PO_2_ (Table 4). Among patients with SPHS, on admission Hb was 10.1 (SD 1.6) compared to 11.2 gdL (SD 4.1) among those without SPHS (*t* = 1.543, *p*=. 126).

**Table 4:** Blood biochemistry of patients with leptospirosis associated SPHS

	Median	Inter quartile Range 25.0–75.0	
Total white cell count			
On admission	9.6	7.6	12.3
Before 1st haemorrhage	11.4	8.6	12.8
After 1st haemorrhage	11.1	8.5	15.2
Platelet Count			
Minimum	36.5	21.0	68.8
On admission	51.0	21.3	105.3
Before 1st haemorrhage	52.0	33.5	86.0
After 1st haemorrhage	56.0	29.5	95.0
Serum Creatinine (mmol/L)			
On admission	261.0	146.0	411.0
Before 1st haemorrhage	260.0	155.0	476.0
After 1st haemorrhage	240.5	135.8	466.5
AST (IU/L)			
On admission	65.5	39.8	120.3
Before 1st haemorrhage	93.0	43.3	408.0
After 1st haemorrhage	88.0	30.5	815.5
ALT (IU/L)			
On admission	56.0	37.5	102.0
Before 1st haemorrhage	57.5	37.5	84.0
After 1st haemorrhage	59.5	28.8	142.0
Serum Bilirubin (μmol/L)			
On admission	42.1	24.2	72.0
Before 1st haemorrhage	44.6	14.5	93.5
After 1st haemorrhage	37.0	11.0	205.0
pH			
On admission	7.39	7.30	7.45
Before 1st haemorrhage	7.40	7.31	7.47
After 1st haemorrhage	7.43	7.28	7.50
Bicarbonate (mEq/L)			
On admission	18.0	14.0	20.0
Before 1st haemorrhage	18.5	12.6	22.9
After 1st haemorrhage	21.0	16.5	25.0
PCO_2_			
On admission	26.0	21.4	30.0
Before 1st haemorrhage	32.0	25.0	38.5
After 1st haemorrhage	28.0	23.5	43.0
PO_2_			
On admission	72.0	56.5	86.5
Before 1st haemorrhage	99.5	78.5	100.0
After 1st haemorrhage	76.0	67.0	100.0

### Therapy and response on patients with SPHS

In this group of leptospirosis patients with SPHS, 59 (75.5%) received TPE and 17 of them died (28.8%). Of the 19 leptosprosis patients with SPHS who did not receive TPE, 17 died (89.5%) (Chi-square 21.507, *p* < .001). Further analysis of TPE and mortality by duration of hospital stay, showed that of the 34 deaths, 19 were within the first 3 days of admission (On the same day 8, and within next 48 h 11). In only four of them was there sufficient time to plan for and for them to receive TPE before death, thus not comparable with the patients survived. The same observation was made for intravenous immunoglobulin (IVIG) (Table 5), where the patient who died without treatment were within first 2 days.

**Table 5:** Mortality / survival associated with different interventions; patients with SPHS

Intervention	Died		Survived	
	n	%	n	%
No specific intervention	16	100	0	0
TPE & IVIG	10	24.4	31	75.6
TPE only	7	38.9	11	61.1
IVIG only	1	33.3	2	66.7

*TPE* therapeutic plasma exchange, *IVIG* intravenous immunoglobulin

The survival rate was higher (*n* = 35, 74.5%) when the TPE was performed within the first 48 h of detecting LPH compared to patients in whom the procedure was done after 48 h (*n* = 5, 54.5%). However, this difference, was not significant (Chi square, P value .191) (Table 6).

**Table 6:** Mortality / survival associated with different times of intervention with TPE

Time from detection of SPHS to intervention with TPE	Survived		Dead	
	N	%	n	%
Within first 48 h	35	74.5	12	25.5
> 48 h	6	54.5	5	45.5

*SPHS* leptospirosis associated severe pulmonary haemorrhagic syndrome, *TPE* therapeutic plasma exchange

Potential factors associated with SPHS were analyzed and several showed significant associations (Table 7).

**Table 7:** Socio-demographic, clinical and life style associated potential risk factors

Parameter		SPHS		No LPH		*P* value^*^
		n	%	N	%	
Age	< 40 years	28	59.6	19	40.4	0.7053
	> 40 years	52	64.2	29	35.8	
Sex	Male	68	63.6	39	36.4	0.6263
	Female	12	57.1	9	42.9	
Smoking	Yes	37	100.0	0	0.0	0.0091
	No	43	82.7	9	17.3	
Alcohol consumption	Yes	41	93.2	3	6.8	0.4845
	No	39	86.7	6	13.3	
Oliguria on admission	Yes	71	71.0	29	29.0	0.0003
	No	9	32.1	19	67.9	
Conjunctival injections	Yes	54	76.1	17	23.9	0.0005
	No	26	45.6	31	54.4	
Tachycardia (> 100 bpm)	Yes	70	69.3	31	30.7	0.0033
	No	10	37.0	17	63.0	
Hypotension	Yes	62	81.6	14	18.4	< 0.0001
	No	18	34.6	34	65.4	
Tachypnoea	Yes	72	80.9	17	19.1	< 0.0001
	No	8	20.5	31	79.5	
Arterial hypoxaemia	Yes	68	94.4	4	5.6	< 0.0001
	No	12	21.4	44	78.6	
Leukocytosis	Yes	69	75.0	23	25.0	< 0.0001
	No	11	30.6	25	69.4	
Thrombocytopenia	Yes	54	84.4	10	15.6	< 0.0001
	No	26	40.6	38	59.4	

^*^Chi-square test or Fishers exact test

## Discussion

In this study, complications were observed in nearly 90% of hospitalized patients with clinically and serologically proven leptospirosis. A majority had AKI, whilst pulmonary, liver and cardiac involvement was noted in the others. A recent systematic review by Warnasekara and colleagues reported that pulmonary involvement in leptospirosis reported from Sri Lanka may range from 1.4 to 45.4% [19]. In comparison to this systematic review, we observed the highest reported pulmonary involvement. Although this may seems in keeping with similar patterns of increasing SPHS reported in the recent past [3, 27], it could be due to the fact that there was a selection bias. Since the plasma exchange prgramme was initiated, more cases of SPHS were tested for leptospirosis from this hospital. Moderate leptospirosis patient without severe complications are not routinely tested in the internal medicine wards. Though the proportion may not reflect actual proportions, we report here the largest cohort of SPHS associated with leptospirosis reported in Sri Lanka.

The reason for this high proportion of SPHS in our study sample compared to other studies reported from Sri Lanka is still not clear. Though the increasing awareness is contributing to increase detection, the large number of patients reported in this study with SPHS could not be attributed totally to increased awareness. Several recent reports from Sri Lanka showed that the different outbreaks of leptospirosis are associated with different clinical presentations such as pancreatitis [28] and cardiac involvement [29]. Further, regional differences of leptospirosis in Sri Lanka are partly explained with infecting *Leptospira* [30]. Our study did not examine the infecting *Leptospira* and further studies on SPHS with identification of infecting serotypes/genotypes are required.

We found several factors to be associated with pulmonary haemorrhage in patients with leptospirosis. However, given the nature of this study, it is difficult to comment if these are actual predictors or early manifestations of SPHS. The interpretation is further complicated by the finding that a majority of patients had multiple organ involvement. Future studies should explore the ability of these factors to their ability either individually or collectively predict SPHS. Currently there is no reliable method to detect high risk patients and a detailed clinical history, clinical examination and close monitoring are used for early detection of complications. We observed unexplained persistent hypotension despite volume correction, unexplained arterial hypoxia and persistent tachycardia to precede the onset of complications specially that of SPHS. It is unclear whether these abnormalities are a reflection of severe sepsis or early radiologically undetected SPHS. Tachypnoea was another factor found to be strongly associated with SPHS. Patients with high risk factors need careful monitoring for early detection of pulmonary haemorrhage and perhaps early transfer to a tertiary care center for specialized care. A previous study found hemodynamic instability (shock within the first 24 h), altered mental status (GCS score < 15), serum potassium, serum creatinine and respiratory rate to be risk factors of SPHS.

SPHS is possibly immune mediated; hence it is logical to suppress dysregulated immune responses prior to the onset of immune-mediated tissue damage. We observed a “possible” better survival rate among patients who received TPE in addition to the standard therapy. There was no significant mortality benefit by adding IVIG to patients already receiving TPE. In 2010, Trivedi et al. also found mortality benefit in patients who received TPE in addition to standard treatment [14]. A few case reports also points towards the beneficial effects of TPE in patients with SPHS [13, 31]. Ours was an observational study and was not designed to assess the efficacy of TPE in patients with SPHS. Decision to offer TPE was taken by specialist clinicians based on their experience, ability of patients to undergo such procedure and the availability of resources. Hence the two groups were not comparable or prognostically similar at baseline. This may be the reason for duration-disaggregated data showing no difference in therapy. However, our observations provide the basis for conducting randomized controlled clinical trials on the efficacy of TPE.

This study has a few limitations. This was an observational study with a relatively small number of patients. There was no uniformity in the management of SPHS in different medical units. Even in the same unit, SPHS patients were managed in different ways at different times based on availability of resources and patient related factors such as cardiovascular stability and comorbidity.

## Conclusions

This study shows a relatively higher percentage of SPHS among clinically and serologically confirmed leptospirosis patients in Southern Sri Lanka. Despite receiving care in a tertiary care unit, the death rate was very high. Clinical studies and therapeutic trials including the assessment of possible role of therapeutic plasma exchange is required to bring down the deaths due to leptospirosis pulmonary haemorrhage.

## Abbreviations

AKI: Acute kidney injury
GC: Glucocorticoids
IVIG: Intravenous immunoglobulin
MAT: Microscopic Agglutination Test
MOH: Medical Officer of Health
RRT: Renal replacement therapy
SPHS: Severe pulmonary haemorrhagic syndrome
THK: Teaching Hospital-Karapitiya
TPE: Therapeutic plasma exchange

## References

[1] Costa F, Hagan JE, Calcagno J, Kane M, Torgerson P, Martinez-Silveira MS, Stein C, Abela-Ridder B, Ko AI (2015). Global morbidity and mortality of leptospirosis: a systematic review. PLoS Negl Trop Dis.

[2] Taylor AJ, Paris DH, Newton PN (2015). A systematic review of the mortality from untreated leptospirosis. PLoS Negl Trop Dis.

[3] Gouveia EL, Metcalfe J, de Carvalho AL, Aires TS, Villasboas-Bisneto JC, Queirroz A, Santos AC, Salgado K, Reis MG, Ko AI (2008). Leptospirosis-associated severe pulmonary hemorrhagic syndrome, Salvador, Brazil. Emerg Infect Dis.

[4] Trevejo RT, Rigau-Perez JG, Ashford DA, McClure EM, Jarquin-Gonzalez C, Amador JJ, de los Reyes JO, Gonzalez A, Zaki SR, Shieh WJ (1998). Epidemic leptospirosis associated with pulmonary hemorrhage-Nicaragua, 1995. J Infect Dis.

[5] Seijo A, Coto H, San Juan J, Videla J, Deodato B, Cernigoi B, Messina OG, Collia O, de Bassadoni D, Schtirbu R (2002). Lethal Leptospiral pulmonary hemorrhage: an emerging disease in Buenos Aires, Argentina. Emerg Infect Dis.

[6] Gulati S, Gulati A (2012). Pulmonary manifestations of leptospirosis. Lung India.

[7] Edwards CN, Nicholson GD, Hassell TA, Everard COR, Callender J (1986). Thrombocytopenia in leptospirosis: the absence of evidence for disseminated intravascular coagulation. Am J Trop Med Hyg.

[8] Nally JE, Chantranuwat C, Wu XY, Fishbein MC, Pereira MM, Da Silva JJ, Blanco DR, Lovett MA (2004). Alveolar septal deposition of immunoglobulin and complement parallels pulmonary hemorrhage in a Guinea pig model of severe pulmonary leptospirosis. Am J Pathol.

[9] Croda J, Neto AN, Brasil RA, Pagliari C, Nicodemo AC, Duarte MI. Leptospirosis pulmonary haemorrhage syndrome is associated with linear deposition of immunoglobulin and complement on the alveolar surface. Clin Microbiol Infect. 2009. 10.1111/j.1469-0691.2009.02916.x 19778300

[10] Silva JJ, Dalston MO, Carvalho JE, Setubal S, Oliveira JM, Pereira MM (2002). Clinicopathological and immunohistochemical features of the severe pulmonary form of leptospirosis. Rev Soc Bras Med Trop.

[11] Yang GG, Hsu YH (2005). Nitric oxide production and immunoglobulin deposition in leptospiral hemorrhagic respiratory failure. J Formos Med Assoc.

[12] Shenoy VV, Nagar VS, Chowdhury AA, Bhalgat PS, Juvale NI (2006). Pulmonary leptospirosis: an excellent response to bolus methylprednisolone. Postgrad Med J.

[13] Meaudre E, Asencio Y, Montcriol A, Martinaud C, Graffin B, Palmier B, Goutorbe P (2008). Immunomodulation in severe leptospirosis with multiple organ failure: plasma exchange, intravenous immunoglobulin or corticosteroids?. Ann Fr Anesth Reanim.

[14] Trivedi SV, Vasava AH, Bhatia LC, Patel TC, Patel NK, Patel NT (2010). Plasma exchange with immunosuppression in pulmonary alveolar haemorrhage due to leptospirosis. Indian J Med Res.

[15] Cerdas-Quesada C (2011). Potential benefits of plasma exchange by apheresis on the treatment of severe icteric leptospirosis: case report and literature review. Transfus Apher Sci.

[16] Dursun B, Bostan F, Artac M, Varan HI, Suleymanlar G (2007). Severe pulmonary haemorrhage accompanying hepatorenal failure in fulminant leptospirosis. Int J Clin Pract.

[17] Agampodi S, Peacock SJ, Thevanesam V (2009). The potential emergence of leptospirosis in Sri Lanka. Lancet Infect Dis.

[18] Warnasekara J. N., Agampodi S. (2017). Leptospirosis in Sri Lanka. Sri Lankan Journal of Infectious Diseases.

[19] Warnasekara J, Koralegedara I, Agampodi S (2019). Estimating the burden of leptospirosis in Sri Lanka; a systematic review. BMC Infect Dis.

[20] Ruwanpura R, Rathnaweera A, Hettiarachchi M, Dhahanayake K, Amararatne S (2012). Severe pulmonary leptospirois associated with high fatality rate: an autopsy series in Galle, southern Sri Lanka. Med J Malaysia.

[21] Herath NJ, Uluwattage WH, Walivita T, Karunanayake L, Karunanayake P, Ratnatunga N, Agampodi S, Senevirathne SL, Wijayaratne GB, Madanayake DC, et al. Benefit of Therapeutic Plasma Exchange in Leptospiral Pulmonary Hemorrhage. In: 50th Annual Academic Sessions of Ceylon College of Physicians. Colombo; 2017. p. 140.

[22] Ramachandran S, Perera MV (1977). Cardiac and pulmonary involvement in leptospirosis. Trans R Soc Trop Med Hyg.

[23] Kularatne SA, Budagoda BD, de Alwis VK, Wickramasinghe WM, Bandara JM, Pathirage LP, Gamlath GR, Wijethunga TJ, Jayalath WA, Jayasinghe C (2011). High efficacy of bolus methylprednisolone in severe leptospirosis: a descriptive study in Sri Lanka. Postgrad Med J.

[24] Niloofa R, Fernando N, de Silva NL, Karunanayake L, Wickramasinghe H, Dikmadugoda N, Premawansa G, Wickramasinghe R, de Silva HJ, Premawansa S (2015). Diagnosis of leptospirosis: comparison between microscopic agglutination test, IgM-ELISA and IgM rapid Immunochromatography test. PLoS One.

[25] Reller ME, Bodinayake C, Nagahawatte A, Devasiri V, Kodikara-Arachichi W, Strouse JJ, Flom JE, Dumler JS, Woods CW (2011). Leptospirosis as frequent cause of acute febrile illness in southern Sri Lanka. Emerg Infect Dis.

[26] Khwaja A (2012). KDIGO clinical practice guidelines for acute kidney injury. Nephron Clin Pract.

[27] Seijo A, Coto H, San Juan J, Videla J, Deodato B, Cernigoi B, Garcia Messina O, Collia O, de Bassadoni D, Schtirbu R (2002). Respiratory distress due to pulmonary hemorrhage in leptospirosis. Medicina (B Aires).

[28] Herath NJ, Kamburapola CJ, Agampodi SB (2016). Severe leptospirosis and pancreatitis; a case series from a leptospirosis outbreak in Anuradhapura district, Sri Lanka. BMC Infect Dis.

[29] Jayathilaka P, Mendis ASV, Perera M, Damsiri HMT, Gunaratne AVC, Agampodi SB (2019). An outbreak of leptospirosis with predominant cardiac involvement: a case series. BMC Infect Dis.

[30] Agampodi SB, Dahanayaka NJ, Bandaranayaka AK, Perera M, Priyankara S, Weerawansa P, Matthias MA, Vinetz JM (2014). Regional differences of leptospirosis in Sri Lanka: observations from a flood-associated outbreak in 2011. PLoS Negl Trop Dis.

[31] Tse KC, Yip PS, Hui KM, Li FK, Yuen KY, Lai KN, Chan TM (2002). Potential benefit of plasma exchange in treatment of severe icteric leptospirosis complicated by acute renal failure. Clin Diagn Lab Immunol.

